# Complementary medicine use, views, and experiences: a national survey in England

**DOI:** 10.3399/bjgpopen18X101614

**Published:** 2018-11-14

**Authors:** Debbie Sharp, Ava Lorenc, Richard Morris, Gene Feder, Paul Little, Sandra Hollinghurst, Stewart W Mercer, Hugh MacPherson

**Affiliations:** 1 Professor of Primary Care, Centre for Academic Primary Care, Population Health Sciences, Bristol Medical School, University of Bristol, Bristol, UK; 2 Senior Research Associate, Centre for Academic Primary Care, Population Health Sciences, Bristol Medical School, University of Bristol, Bristol, UK; 3 Professor in Medical Statistics, Centre for Academic Primary Care, Population Health Sciences, Bristol Medical School, University of Bristol, Bristol, UK; 4 Professor of Primary Care, Centre for Academic Primary Care, Population Health Sciences, Bristol Medical School, University of Bristol, Bristol, UK; 5 Professor of Primary Care Research, Primary Medical Care, Faculty of Medicine, University of Southampton, Southampton, UK; 6 Senior Lecturer in Health Economics, Centre for Academic Primary Care, Population Health Sciences, Bristol Medical School, University of Bristol, Bristol, UK; 7 Chair in Primary Care Research, Primary Care and General Practice, Institute of Health and Wellbeing, University of Glasgow, Glasgow, UK; 8 Professor of Acupuncture Research, Department of Health Sciences, University of York, York, UK

**Keywords:** Complementary Therapies, Surveys and Questionnaires, General Practice

## Abstract

**Background:**

In 2005,12% of the English population visited a complementary and alternative medicine (CAM) practitioner.

**Aim:**

To obtain up-to-date general population figures for practitioner-led CAM use in England, and to discover people's views and experiences regarding access.

**Design & setting:**

A face-to-face questionnaire survey was commissioned. A nationally representative adult quota sample (aged ≥15 years) was used.

**Method:**

Ten questions were included within Ipsos MORI’s weekly population-based survey. The questions explored 12-month practitioner-led CAM use, reasons for non-use, views on NHS-provided CAM, and willingness to pay.

**Results:**

Of 4862 adults surveyed, 766 (16%) had seen a CAM practitioner. People most commonly visited CAM practitioners for manual therapies (massage, osteopathy, chiropractic) and acupuncture, as well as yoga, pilates, reflexology, and mindfulness or meditation. Women, people with higher socioeconomic status (SES) and those in south England were more likely to access CAM. Musculoskeletal conditions (mainly back pain) accounted for 68% of use, and mental health 12%. Most was through self-referral (70%) and self-financing. GPs (17%) or NHS professionals (4%) referred and/or recommended CAM to users. These CAM users were more often unemployed, with lower income and social grade, and receiving NHS-funded CAM. Responders were willing to pay varying amounts for CAM; 22% would not pay anything. Almost two in five responders felt NHS funding and GP referral and/or endorsement would increase their CAM use.

**Conclusion:**

CAM use in England is common for musculoskeletal and mental health problems, but varies by sex, geography, and SES. It is mainly self-referred and self-financed; some is GP-endorsed and/or referred, especially for individuals of lower SES. Researchers, patients, and commissioners should collaborate to research the effectiveness and cost-effectiveness of CAM and consider its availability on the NHS.

## How this fits in

Figures from 2005 reported that 12% of the English population used practitioner-led CAM. This 2015 survey has found that 16% of the general population had used practitioner-led CAM in the previous 12 months. Most CAM use is self-referred, for musculoskeletal problems, particularly by women and those of higher SES, although some is GP-endorsed and/or referred, for individuals of lower SES. Researchers, patients, and commissioners should collaborate to research the effectiveness and cost-effectiveness of CAM and consider its availability on the NHS.

## Introduction

CAM is 'a diverse group of health-related therapies and disciplines which are not considered to be a part of mainstream medical care' and includes osteopathy, chiropractic, acupuncture, herbal medicine, and homeopathy.^1^ This survey focused on practitioner-led CAM, for which the prevalence was 10% in 2001 (UK),^2^ and 12% in 2005 (England).^3^ If over-the-counter and/or self-care CAM are included, 12-month prevalence estimates of CAM use in Europe range from 0.3–86%;^4^ recent UK adult annual CAM use estimates were 28% (England only),^5^ 20%,^6^ and 26% (England only)^3^ in 1998, 1999, and 2005 respectively. A systematic review reported a 41% average 1-year prevalence CAM use in the UK and 52% average lifetime prevalence.^7^ There has not been a national English survey since 2005.

The evidence base for CAM varies widely. For musculoskeletal and mental health conditions, which are common in primary care, and were the focus of the authors' larger scoping study,^8^ good quality reviews were identified with moderate to good quality evidence of effectiveness for yoga,^9^ osteopathy,^10^ acupuncture,^11,12^ and spinal manipulation and/or mobilisation^12–14^ for low back pain; acupuncture for myofascial trigger point pain;^15^ tai chi^16^ and acupuncture^17,18^ for osteoarthritis; manual therapy,^19^ manipulation,^20^ and acupuncture^12^ for neck pain; acupuncture for fibromyalgia;^21^ mindfulness and/or meditation,^22,23^ and tai chi^24^ for depression;^25^ meditation and/or mindfulness-based stress reduction for anxiety;^22,26^ meditative and/or mind-body movement for sleep;^27^ and mindfulness for stress and distress.^22^

There is little up-to-date information on current NHS provision of CAM. A recent survey suggests that a million NHS acupuncture appointments are provided annually.^28^ A systematic review of surveys estimated that >20% of UK physicians had 'used' CAM in their practice within the previous week (recommendations, referrals, provision of treatment, or self-administration), an average of 39% of physicians had referred to and 46% had recommended CAM.^7^ A 2003 article found around half of general practices offered some CAM services, mainly acupuncture, osteopathy, and homeopathy.^29^ In 2009, 19% of GPs were regularly treating patients with CAM; 56% had treated with, referred to, or endorsed CAM during the previous week.^30^

Given the lack of recent data on CAM use by the public or NHS CAM access, and in the light of the emphasis in the 2014 NHS Five Year Forward View on self-care, prevention, and wellbeing,^31^ the authors conducted a national survey with Ipsos MORI of the public’s use of CAM and their views on CAM provision within the NHS.

## Method

The aim of the survey was to obtain up-to-date figures on CAM use in the general population in England, and explore views and experiences regarding consulting CAM practitioners. The specific objectives were:

1. to identify the proportion of the population in England that had used practitioner-led CAM in the previous year, and explore reasons for non-use;
2. to identify the health conditions most commonly treated with CAM;
3. to identify how CAM is accessed and funded, and how much it costs individuals; and
4. to obtain the public’s views on models of CAM provision within the NHS and willingness to pay for CAM.

The authors commissioned Ipsos MORI (a UK market research company) to include 10 questions within their national, weekly 'Capibus' survey (https://www.ipsos-mori.com/ourexpertise/omnibusservices/capibus.aspx). This face-to-face computer-assisted survey, completed in people’s homes, used a quota sample of all adults (aged ≥15 years) in England. Quota sampling aims for a nationally representative sample of adults in England, based on age, sex, and working status, within four regions (South, North, Midlands, and London). The survey ran between 25 September and 18 October 2015.

The project team developed 10 survey questions (see Appendix) with input from Ipsos MORI, the project patient and public involvement group, and steering group. The questions included: whether practitioner-based complementary medicine had been used during the previous 12 months; reason for use; access; funding; cost; reasons for non-use; views on particular models of NHS CAM provision that might facilitate its use; and willingness to pay. Ipsos MORI provided basic demographic data. The authors combined income, social grade, and employment into SES. The authors defined CAM by showing participants a list of practitioners, including NHS professionals delivering CAM (for example, GP, physiotherapist, or nurse), adapted from that used by the National Library of Medicine's MeSH term 'Complementary therapies'.^32^ The list included most of the CAM included by Thomas and Hunt^2,3^ (but excluding crystal healing, dowsing, iridology, kinesiology, relaxation; adding art therapy, Bach flowers, biofeedback, craniosacral, emotional freedom technique, Feldenkrais, guided imagery, music therapy, pilates, qi gong, tai chi, Trager, and yoga). An open-ended question asked what help they were seeking from the CAM practitioner; free-text responses were coded.

Data were weighted by Ipsos MORI for the English adult population using region, social grade, age, working status, housing tenure, and ethnicity (white or black and minority ethnic), based on census data or mid-year estimates and National Readership Survey defined profiles. Analyses were mainly descriptive; statistical testing compared different groups. In addition to the univariable analyses, a multivariable analysis using logistic regression was conducted to test whether bivariable associations were independent of each other, estimating odds ratios with 95% confidence intervals. Not all aspects of CAM use were amenable to multivariable analysis; the authors excluded those with events less than 10 times the number of parameter estimates required in any specific model.^33^

## Results

The final sample comprised 4862 adults (response rates unavailable). See Table 1 for participant characteristics.

**Table 1:** Participant characteristics (*n* = 4862)

	*n*	%
**Sex**		
Male	2378	49
Female	2484	51
Missing data	0	0
**Age (years)**		
15–24	762	16
25–34	817	17
35–44	771	16
45–54	832	17
55–64	656	13
65+	1024	21
Missing data	0	0
**Social grade (based on occupation of chief income earner)^a^**		
A	168	3
B	1163	24
C1	1317	27
C2	1053	22
D	744	15
E	416	9
Missing data	1	<1
**Household income (annual)**		
Up to £11 499	650	13
£11 500–£24 999	804	17
£25 000+	1664	34
Missing data	1744	36^b^
**Marital status**		
Married/living as	2801	58
Single	1407	29
Widowed/divorced/separated	636	13
Missing data	18	<1
**Working status**		
Working	2676	55
In education	398	8
Not working	1788	37
Missing data	0	0
**Region of England**		
North	1395	29
Midlands	1057	22
South	1697	35
London	713	15
Missing data	0	0
**Ethnicity**		
White	4171	86
Mixed	46	1
Asian	452	9
Black	130	3
Other	53	1
Missing data	10	<1
**Housing tenure**		
Mortgage/owned	3028	62
Rented	1751	36
Other	38	1
Missing data	45	1
^a^Based on National Readership Survey categories: A: High managerial, administrative, or professional B: Intermediate managerial, administrative, or professional C1: Supervisory, clerical and junior managerial, administrative, or professional C2: Skilled manual workers D: Semi and unskilled manual workers E: State pensioners, casual, or lowest grade workers, unemployed with state benefits only ^b^Missing data of around one-third is usual for the Capibus survey		

### CAM use

A total of 766 participants (16% of sample) had seen a CAM practitioner (from the list) in the previous 12 months. Table 2 shows manual therapies were most common. The main route to a CAM practitioner was self-referral (*n* = 539, 70%), followed by GP referral or recommendation (*n* = 133, 17%). The majority of responders (67%) paid in full for their CAM but, for 13% of CAM users, the NHS paid (the remaining 20% selected other options, such as 'another organisation such as a charity paid for all of it'). 'GP or NHS health professional-provided CAM' included physiotherapist-delivered CAM, 'traditional medicine', tai chi, yoga, osteopathy, acupuncture, and meditation and/or mindfulness.

**Table 2:** Characteristics of CAM use (*n* = 766)

	*n*	%
**Type of CAM practitioner**		
Massage practitioner	143	19
Osteopath	91	12
Acupuncturist	88	11
Chiropractor	87	11
Yoga teacher	52	7
Physiotherapist-delivered CAM	41	5
Pilates teacher	28	4
Reflexologist	22	3
Meditation and/or mindfulness teacher	20	3
Homeopath	20	3
Reiki practitioner	17	2
Hypnotherapist	15	2
Herbalist	14	2
Chinese herbal medical practitioner	12	2
Other	74	10
**Number of times they saw the CAM practitioner in the past 12 months**		
Once a year	185	24
2–3 times	211	28
4–6 times	177	23
Once or twice a month	115	15
Once or twice a week	68	9
More than twice a week	8	1
Don’t know	2	<1
**Route to CAM practitioner**		
I found them myself or they were recommended by friend/family	539	70
My GP referred or recommended me	133	17
Another NHS health professional (for example consultant) referred me	33	4
Another complementary practitioner referred or recommended me	33	4
My GP or another NHS health professional I was seeing provided the complementary treatment themselves	14	2
Through company or work insurance	11	1
Other	22	3
**Payment for treatment by CAM practitioners**		
I or my family/friend paid for all of it	510	67
The NHS paid for all of it	103	13
I or my family/friend paid for part of it	50	7
My health insurance paid for all of it	22	3
Free (general)	16	2
The NHS paid for part of it	15	2
Another organisation, such as a charity, paid for all of it	14	2
Other	41	5
Don’t know	2	<1
**Cost per visit to the CAM practitioner^a^**		
Less than £10	76	14
Between £11 and £20	55	10
Between £21 and £30	126	23
Between £31 and £40	163	29
Between £41 and £50	73	13
Between £51 and £60	35	6
Between £60 and £100	23	4
Over £100	5	1
Prefer not to say/don’t know	3	<1
^a^This only applies to the *n* = 560 who paid for all or part of the CAM themselves		

Table 3 shows univariable and multivariable demographic associations. CAM use was associated with being female, higher SES (higher social grade, higher household income, being employed) and living in the south of England (all *P*<0.001). Remarkably, few associations changed in the multivariable analysis. Only marital status and housing status were not independently associated with CAM use.

**Table 3:** Univariable and multivariable associations of overall CAM use

		Univariable analysis		Multivariable analysis	
**Factor**	**Category**	**Odds ratio (95% CI)**	***P* value**	**Odds ratio (95% CI)**	***P* value**
Sex	Male	1		1	
	Female	1.74 (1.48 to 2.04)	<0.001	1.79 (1.50 to 2.13)	<0.001
Age	15–24	1		1	
	25–34	1.75 (1.28 to 2.4)	<0.001	1.81 (1.24 to 2.65)	0.002
	35–44	2.74 (2.02 to 3.71)	<0.001	2.50 (1.70 to 3.69)	<0.001
	45–54	2.47 (1.83 to 3.34)	<0.001	2.04 (1.37 to 3.03)	<0.001
	55–64	2.29 (1.67 to 3.14)	<0.001	2.09 (1.38 to 3.15)	<0.001
	65+	1.48 (1.09 to 2.02)	0.012	1.49 (0.95 to 2.34)	0.081
Ethnicity	White	1		1	
	Mixed	1.49 (0.75 to 2.98)	0.26	1.73 (0.93 to 3.24)	0.085
	Asian	0.35 (0.24 to 0.51)	<0.001	0.46 (0.33 to 0.66)	<0.001
	Black	0.44 (0.23 to 0.82)	0.01	0.56 (0.31 to 0.99)	0.047
	Other	0.95 (0.46 to 1.98)	0.89	0.78 (0.36 to 1.68)	0.52
Housing tenure	Owner			1	
	Rented	0.61 (0.51 to 0.72)	<0.001	0.84 (0.68 to 1.04)	0.11
Income	Low (up to £11 499)	1		Not conducted, due to 30% missing data	
	Medium (£11 500–£24 999)	1.13 (0.83 to 1.52)	0.43		
	High (£25 000 or more)	1.78 (1.38 to 2.31)	<0.001		
Marital status	Married	1		1	
	Single	0.72 (0.6 to 0.86)	<0.001	1.21 (0.96 to 1.52)	0.11
	Widowed/divorced/separated	0.83 (0.65 to 1.05)	0.12	1.04 (0.79 to 1.38)	0.77
Region	North	1			
	Midlands	1.07 (0.85 to 1.35)	0.55	1.09 (0.86 to 1.37)	0.47
	South	1.69 (1.4 to 2.06)	<0.001	1.46 (1.18 to 1.81)	<0.001
	London	0.83 (0.63 to 1.1)	0.19	0.90 (0.68 to 1.20)	0.49
Working status	Employed	1		1	
	Student	0.48 (0.34 to 0.68)	<0.001	0.89 (0.59 to 1.35)	0.60
	Unemployed	0.65 (0.55 to 0.77)	<0.001	0.74 (0.59 to 0.92)	0.008
Social grade	(per grade)	0.77 (0.73 to 0.82)	<0.001	0.82 (0.76 to 0.88)	<0.001

Musculoskeletal conditions, mainly back pain, accounted for 68% of CAM use (see Table 4). Men and those aged >65 years were more likely to cite musculoskeletal conditions, the latter mainly for arthritis. Musculoskeletal conditions were significantly more likely to be treated with massage, osteopathy, and chiropractic than were mental health conditions, which were more likely to be treated with meditation and/or mindfulness or reiki. Eleven percent of CAM use was non-condition-specific, that is for prevention or general health. Women were more likely to use CAM for arthritis and minor mental health conditions. The majority (*n* = 632) used CAM for one condition; *n* = 101 used it for two conditions; *n* = 25 for three conditions; *n* = 6 for four conditions; *n* = 1 for five conditions; and *n* = 1 for six conditions.

**Table 4:** Health conditions treated by a CAM practitioner (*n* = 766)

	*n*	%
Musculoskeletal (net)	520	68
Back pain	292	38
Other musculoskeletal pain (neck pain, shoulder pain, knee pain)	172	22
Arthritis (osteo- or rheumatoid)	48	6
Headaches/migraines	33	4
Other chronic pain	29	4
Fibromyalgia	10	1
Other (net)	223	2
Women's health	25	3
Preventative	23	3
Relaxation	23	3
General health/wellbeing	22	3
Exercise/keeping fit	17	2
Digestive problems	13	2
Other	124	16
Mental health (net)	92	12
Minor mental health symptoms (minor depression, anxiety, stress)	50	7
Tiredness or fatigue	22	3
Sleep problems/insomnia	15	2
Serious mental health conditions	9	1
Chronic fatigue syndrome/myalgic encephalomyelitis	8	1
Don’t know	4	1
No answer	6	1

Table 5 shows the pathways to (GP referred and/or recommended, or self-referred) and payment (NHS or not) for the most popular CAM. The most commonly used CAM therapies (shown in Table 5) were similar across all four categories.

**Table 5:** Pathways to and payment for practitioners offering 16 most popular CAM therapies

	GP referred/ recommended (*n *= 133)		Self-referred (*n *= 539)		*P* value	NHS paid for some or all (*n* = 117)		NHS did not pay (*n* = 649)		*P* value
	*n*	**%** (of column)	*n*	**%** (of column)		*n*	**%** (of column)	*n*	**%** (of column)	
Acupuncture^a^	22	16	52	10	0.021	21	29	67	12	0.017
Physiotherapist-delivered CAM^ab^	20	15	15	3	<0.001	15	21	26	5	<0.001
Chiropractic^a^	13	10	67	13	0.42	5	7	82	14	0.009
Osteopathy	11	8	75	14	0.09	10	14	80	14	0.28
Massage^a^	9	6	110	20	<0.001	10	14	133	23	0.002
Yoga^a^	4	3	45	8	0.036	2	3	50	9	0.015
Pilates	3	2	22	4	0.34	1	1	27	5	0.11
Reflexology	1	1	19	4	0.15	1	1	21	4	0.23
Homeopathy	1	1	17	3	0.13	0	0	20	4	0.057
Meditation/ mindfulness	4	3	12	2	0.58	4	6	16	3	0.53
Reiki	2	1	13	2	0.74	1	1	16	3	0.49
Hypnotherapy	2	1	12	2	0.62	1	1	14	2	0.71
Herbal medicine	3	2	10	2	0.74	1	1	13	2	0.39
Chinese herbal medicine	1	1	11	2	0.48	1	1	11	2	0.50
Traditional medicine^ac^	6	5	3	1	0.003	9	8	2	>1	<0.001
Nutritional therapy	3	2	3	1	0.09	3	3	7	1	0.19

^a^Statistically significant. ^b^Not included in the list of CAM presented as part of the questionnaire but coded from open responses to Q2

^c^May have been interpreted by participants as meaning conventional medicine.

Table 6 shows that GP-referred and/or recommended users were more likely to be aged <24 years or >55 years, of lower SES, living in the north of England, renting, and Asian. Apart from age, all these demographic associations were reversed for self-referred CAM users. GP-referred and/or recommended users were more likely to have had their CAM paid for by the NHS, although 27% of this subgroup, had paid something towards the CAM. The condition being treated was not associated with the route to CAM or NHS payment.

**Table 6:** Cross tabulation of demographics and access route to CAM practitioners

Demographics		Access route to CAM				Unadjusted			Adjusted		
		GP referred/ recommended (*n* = 133)		Self-referred (*n* = 539)		Odds ratio for GP referral	95% confidence interval	*P *value	Odds ratio for GP referral	95% confidence interval	*P *value
		*n*	% of row	*n*	% of row						
Sex	Male	55	19	186	65	1.00			1.00		
	Female	78	16	353	74	0.81	0.56 to 1.19	0.286	0.69	0.44 to 1.07	0.097
Age	15–24	14	21	42	61	1.00			1.00		
	25–34	15	13	86	72	0.56	0.25 to 1.23	0.147	0.62	0.25 to 1.51	0.293
	35–44	16	10	120	73	0.41	0.19 to 0.88	0.023	0.48	0.19 to 1.22	0.124
	45–54	25	15	118	72	0.69	0.34 to 1.42	0.315	0.91	0.36 to 2.30	0.849
	55–64	28	23	84	69	1.13	0.55 to 2.32	0.741	1.37	0.54 to 3.46	0.51
	65+	34	26	89	68	1.34	0.66 to 2.70	0.417	1.15	0.41 to 3.20	0.796
Ethnicity	White	119	17	501	71	1.00			1.00		
	Asian	1	12	8	74	0.64	0.10 to 4.26	0.646	0.83	0.16 to 4.37	0.826
	Black	10	34	15	50	2.47	1.13 to 5.42	0.024	2.73	1.25 to 5.98	0.012
	Mixed	2	18	6	57	1.08	0.22 to 5.23	0.928	1.27	0.29 to 5.54	0.752
	Other	1	6	8	87	0.31	0.02 to 5.25	0.414	0.37	0.04 to 3.65	0.398
Housing tenure	Mortgaged	79	14	413	75	1.00			1.00		
	Rented	51	2	122	59	1.90	1.28 to 2.83	0.001	2.43	1.47 to 4.03	0.001
	Other	2	25	5	50	1.94	0.42 to 9.01	0.399	2.17	0.37 to 12.87	0.394
Income	Up to £11 499	25	30	48	56	1.00			1.00		
	£11 500–£24 999	25	20	85	71	0.59	0.31 to 1.12	0.108	Not conducted, due to 30% missing data		
	£25 000+	46	13	266	74	0.33	0.19 to 0.57	<0.001			
Marital status	Married	77	16	346	71	1.00			1.00		
	Single	35	19	125	68	1.25	0.81 to 1.94	0.317	1.03	0.59 to 1.78	0.922
	Widow/divorced/separated	20	21	68	72	1.45	0.84 to 2.51	0.186	1.13	0.59 to 2.15	0.718
Region of England	North	42	22	120	65	1.00			1.00		
	Midlands	23	16	103	69	0.64	0.36 to 1.12	0.115	0.61	0.34 to 1.07	0.086
	South	59	17	251	72	0.70	0.45 to 1.09	0.117	0.82	0.49 to 1.37	0.444
	London	9	11	65	80	0.42	0.19 to 0.92	0.03	0.51	0.23 to 1.14	0.101
Working status	Working	62	13	368	74	1.00			1.00		
	In education	7	17	26	67	1.43	0.60 to 3.43	0.422	0.74	0.27 to 2.04	0.565
	Not working	64	28	145	63	2.66	1.80 to 3.94	<0.001	1.77	1.02 to 3.08	0.043
Social grade^a^	A	2	6	27	76	1.00			1.00		
	B	34	14	174	72	2.41	0.59 to 9.84	0.221	1.94	0.42 to 9.08	0.399
	C1	38	16	181	75	2.77	0.68 to 11.29	0.154	1.91	0.41 to 8.83	0.409
	C2	21	15	95	68	2.65	0.63 to 11.15	0.183	2.00	0.41 to 9.65	0.388
	D	22	30	45	62	6.38	1.50 to 27.17	0.012	3.52	0.71 to 17.50	0.124
	E	16	43	18	49	11.18	2.47 to 50.54	0.002	4.45	0.83 to 23.88	0.082

^a^Based on National Readership Survey categories:

A: High managerial, administrative, or professional

B: Intermediate managerial, administrative, or professional

C1: Supervisory, clerical and junior managerial, administrative, or professional

C2: Skilled manual workers

D: Semi and unskilled manual workers

E: State pensioners, casual, or lowest grade workers, unemployed with state benefits only

### Non-use of CAM

Not needing any health care was the main reason cited by the majority (63%) of non-users; for 16% it had not occurred to them. Lack of need was more commonly cited by those with higher SES and less commonly by Asian and Black responders. Six percent stated that they did not believe in CAM. Concern about practitioners’ professional regulation or qualifications was more common in social grades A and B. Not being available locally was most commonly mentioned by responders in London.

### Factors relating to CAM use

When given a list of possible models of CAM provision, including funding, and asked: 'If you had a health problem you thought complementary or alternative medicine could help with, which, if any, of the following statements would encourage you to use it?' 39% of responders said they would be more likely to use complementary medicine if it was free (NHS-funded), 35% if their GP mentioned it might help, and 27% if their GP referred them.

In general, NHS-related factors (limitations of NHS care, part NHS-payment, or a CAM practitioner who was an NHS professional or NHS regulated) were more often cited by those who were female, had higher income, had a mortgage, and were employed.

CAM users were more likely to respond: 'If I thought it would enhance the care I was already receiving.' Non-users were more likely to respond: 'If my GP mentioned it might help.' Non-users were also more likely to respond: 'If my GP referred me.' Those who were not using CAM owing to lack of healthcare need were more likely to cite NHS endorsement as encouraging them to use CAM. Those who were not using CAM because 'it hasn’t occurred to me' were more likely to say they would consider CAM if they 'had to wait a long time for NHS care' or 'NHS treatment was not helping'.

### Cost of CAM and willingness to pay

For the 560 CAM users who provided cost data, the majority paid £21–£40 per visit (*n *= 289, 52% of responders), as shown in Table 2. The only demographic association was that those in the south or London were more likely to pay £41–£50 and those in London were more likely to pay £60–£100 than any other region.

Non-users were asked: 'If you had a health problem you thought would improve from seeing a complementary or alternative practitioner, what is the most you would be willing to pay for each visit?' Amounts cited varied widely; 17% cited 'between £11 and £20'; 16% 'between £21 and £30'; and 22% said they would not be willing to pay for it at all. High earners, those in work, and those of higher social grade were willing to pay more.

## Discussion

### Summary

Sixteen percent of the surveyed adult population (aged ≥15 years) in England had seen a CAM practitioner in the last 12 months, mainly for manual therapies and most commonly for musculoskeletal conditions (mainly back pain), followed by mental health. Use was more commonly associated with being female, of higher SES and living in the south of England. Most users paid for the CAM themselves. Although the majority of CAM use was via self-referral, a small proportion was GP referred and/or recommended, for a range of conditions and mainly for acupuncture, physiotherapist-delivered CAM, chiropractic, and osteopathy. GP-referred CAM was more common in lower SES groups and more often paid for by the NHS. Willingness to pay for CAM varied widely; for example, 22% of responders said they would not be willing to pay anything, and higher SES responders were willing to pay more.

### Strengths and limitations

Incorporating questions on CAM within a routine national survey was efficient. The Capibus methodology obtained a nationally representative sample, using face-to-face interviews at people’s homes, rigorous geographical sampling, and quotas for demographic characteristics. Data were comprehensively validated and weighted to correct for minor deficiencies or bias in the sample. Although overall response rates are not available, being part of the routine Capibus survey is likely to have avoided the pro-CAM response bias described for other CAM surveys.^34^

The authors could only include a limited number and length of questions. Some questions had a poor response, which limited sub-group analyses.

As for all Ipsos MORI surveys, interviewers did not guide responders or provide clarification beyond the written questions and prompts. Some responders may have misunderstood the authors' CAM definition, particularly CAM delivered by an NHS practitioner; for example, mindfulness within NHS mental health services. Recall bias may have been present, particularly for specific questions such as the cost of the CAM.

### Comparison with existing literature

Practitioner-led CAM use was about 5% higher than previous national (UK and England) surveys.^2,3^ This may relate to the authors' wider CAM definition, which included 11 more therapies than Hunt *et al*,^3^ or increased CAM use since 2005. Exercise-based CAM (pilates, qi gong, tai chi, yoga), used by up to 7% of users, may account for this study's higher prevalence figure. Only osteopaths and acupuncturists featured in both the present survey's and previous national surveys’ top five most commonly accessed practitioners,^2,3^ although chiropractors and massage practitioners were in the authors' one previous survey. The emphasis on manual therapies (massage, osteopathy, and chiropractic), acupuncture, and yoga in the survey reflects current evidence (see Introduction). Pilates and reflexology were popular but have less evidence. Tai chi has some evidence but was not popular.

CAM was most commonly used for musculoskeletal conditions in a UK survey and an EU review.^4,5^ Using CAM for low back pain and arthritis probably reflects the general prevalence of these conditions,^35–37^ and possibly the evidence base for CAM. Having pain, anxiety, depression, or a long-term condition has been associated with seeing a CAM practitioner^3^ and CAM users may more often have multimorbidity.^38^

This study's demographic associations with CAM use have previously been reported: being female,^3–5,38–40^ higher social grade^2^ and income,^2,38,39^ and being employed.^3^ The first may reflect women’s greater use of health care.^41^ Education is associated with social grade, income, and employment and is a stronger predictor of CAM use than income.^38^

Reasons for non-use of CAM are rarely explored,^42^ but often include lack of need for any health care^42,43^ confirming perceived need as predicting healthcare use.^38^ CAM users may use more health care in general,^39,44^ perhaps owing to chronic health conditions.^44^ Although pro-CAM beliefs, for example spirituality, predict CAM use,^45^ strong anti-CAM beliefs, for example safety concerns or poor availability, rarely predict non-use.^42,43^

Despite the small proportion of GP referred and/or recommended CAM, a significant proportion of GPs (19%) endorse CAM in their practice.^30^ However, decreasing GP referral and/or recommendation from 1999 (38%) to 2009 (19%) was attributed to either increased scepticism or NHS financial pressures.^30^ Acupuncture being a common GP referred and/or recommended CAM may reflect its practice by NHS clinicians.^28^ GPs may have referred to or recommended chiropractic and osteopathy due to their statutory regulation.

Willingness to pay for CAM appeared to be based on ability to pay (that is, working and earning more), and free or low-cost CAM might increase use. Older and lower SES patients perhaps cannot afford CAM (they used GP referred and/or recommended and NHS-funded CAM). CAM is primarily paid for by the patient and is accessed outside NHS care, and is used, therefore, by more affluent groups.^46^ The relationship with income may also be due to availability (less CAM in low-income areas) and accessibility (barriers attending CAM appointments among those with lower-income jobs).^47^

### Implications for practice and research

Future surveys could include larger samples (for subgroup analyses for CAM types), questions on 12-month or 'ever' use, perceived benefit, detail on health conditions, multiple uses of CAM, views on NHS integration, and more detail on willingness to pay.

These findings raise the question as to whether GPs and other NHS professionals should routinely ask patients about CAM use, particularly for back pain and other musculoskeletal conditions.^3^

Ability to pay may be a factor in accessing CAM (indicated by the association of CAM use with higher SES; lower SES responders being more likely to be GP-referred to CAM; and responders stating that they may use more CAM if the NHS provided services, and GPs endorsed and/or referred them). Integration of CAM into the NHS through primary care could promote continuity of care, safety, and balance of power.^48^ An integrative medicine approach includes many of the values recently included in UK health policy documents; for example, Five Year Forward View.^49^ It is patient-centred, as discussed in 2010,^50^ focuses on prevention, and emphasises patient self-management and person- and community-centred approaches to health and wellbeing.^31^ Many of these values underpin social prescribing, which is an increasingly popular model of health care.^51^ There seems to be significant patient demand for CAM^52^ and more holistic approaches,^48^ and a view that CAM may improve patient satisfaction.^53^

However, such NHS endorsement would clearly need to be evidence based. For CAM with sufficient evidence for NHS integration, two models of integration may be possible. In ‘selective incorporation’ NHS staff practise CAM or CAM practitioners work on NHS premises.^54^ In Weise’s integration model, GPs refer to CAM services.^55^ The latter may be more feasible, and could fit into a social prescribing model^56^ or the Professional Standards Authority’s ‘Let’s Work Together’ campaign.^57^ Another model is integrated personal commissioning, which enables people with chronic conditions to control their NHS and/or social care resources and ‘micro-commission’ their care.^58^

Referral to CAM via the NHS also raises cost implications. Few responders were willing to pay the full cost (approximately £60 per visit in the UK) of CAM, and 13% said they would consider CAM if it was partly NHS-funded. Co-payment by patients is a contentious issue but, where the evidence base is good, the NHS may need to respond to a changing, consumerist society.^49,59,60^ There is little published about co-payment for CAM.

Complementary medicine is commonly used in England, particularly for musculoskeletal and mental health problems, and by affluent groups paying privately. However, less well-off people are also being GP-referred for NHS-funded treatments. For CAM with evidence of effectiveness (and cost-effectiveness), those of lower SES may be unable to access potentially useful interventions, and access via GPs may be able to address this inequality. Researchers, patients, and commissioners should collaborate to research the effectiveness and cost-effectiveness of CAM, and consider its availability on the NHS.^34^

## Survey questionnaire

**Table:**

Q1 This survey asks about complementary and alternative healthcare practitioners, for example (but not limited to) acupuncture, massage or yoga. This survey only asks about practitioners you have seen, not products you have bought in a shop or self-care at home. Practitioners include therapists and teachers. **In the past 12 months have you seen a complementary or alternative healthcare practitioner for a health-related problem?** A. Yes B. No C. Don’t know **Please include:** Any of the following whether they are NHS professionals or not (e.g. GP, physiotherapist, nurse). For example: A. Acupuncturist B. Chinese herbal medicine practitioner C. Herbalist D. Homeopath E. Hypnotherapist F. Meditation/mindfulness teacher G. Massage practitioner H. Chiropractor I. Osteopath J. Shiatsu practitioner K. Tai chi teacher L. Pilates teacher M. Yoga teacher N. Alexander technique teacher O. Aroma therapist P. Art therapist Q. Ayurvedic practitioner R. Craniosacral therapist S. Healer (e.g. spiritual healer) T. Music therapist U. Nutritional therapist V. Qi gong teacher W. Reflexologist X. Reiki practitioner Y. Anthroposophic medicine practitioner Z. Bach or other flower remedies practitioner AA. Biofeedback practitioner BB. Emotional freedom technique practitioner CC. Feldenkrais practitioner DD. Guided imagery practitioner EE. Traditional medicine practitioner FF. Trager practitioner GG. Naturopath HH. Unani medicine practitioner Please do not include: A. Breathing exercises B. Colour therapy C. Counselling D. Dance therapy E. Light therapy F. Neurolinguistic Programming (NLP) G. Special Diets (e.g. Vegetarianism) H. Play therapy I. Psychotherapy J. Prayer K. TENS (transcutaneous electrical nerve stimulation) L. Any treatment which did not involve a practitioner e.g. purchased from a shop or self-care
ASK ONLY THOSE WHO CODE A AT Q1 Q2 **What type of practitioner did you see? If you saw more than one, please tell us about the one you have seen the most times in the past 12 months.** A. Acupuncturist B. Chinese herbal medicine practitioner C. Herbalist D. Homeopath E. Hypnotherapist F. Meditation/mindfulness teacher G. Massage practitioner H. Chiropractor I. Osteopath J. Shiatsu practitioner K. Tai chi teacher L. Pilates teacher M. Yoga teacher N. Alexander technique teacher O. Aroma therapist P. Art therapist Q. Ayurvedic practitioner R. Craniosacral therapist S. Healer (e.g. spiritual healer) T. Music therapist U. Nutritional therapist V. Qi gong teacher W. Reflexologist X. Reiki practitioner Y. Anthroposophic medicine practitioner Z. Bach or other flower remedies practitioner AA. Biofeedback practitioner BB. Emotional freedom technique practitioner CC. Feldenkrais practitioner DD. Guided imagery practitioner EE. Traditional medical practitioner FF. Trager practitioner GG. Naturopath HH. Unani medicine practitioner II. Other (please specify) JJ. Don’t know
ASK ONLY THOSE WHO CODE A AT Q1 Q3 **And how many times over the past 12 months did you see this complementary practitioner?** A. About once in the past 12 months B. About two or three times in the past 12 months C. About four to six times in the past 12 months D. About once or twice per month E. About once or twice per week F. More than twice a week G. Don’t know
ASK ONLY THOSE WHO CODE A AT Q1 Q4 **What were you seeking help for from this complementary practitioner? You can give more than one reason.** Musculoskeletal/pain A. Back pain B. Arthritis (osteoarthritis or rheumatoid arthritis) C. Fibromyalgia D. Other musculoskeletal pain e.g. neck pain, shoulder pain, knee pain E. Headaches or migraines F. Other chronic pain Mental health G. Minor mental health symptoms e.g. minor depression, anxiety, stress, H. Serious mental health conditions e.g. major depression, schizophrenia, bipolar I. Sleep problems or insomnia J. Tiredness or fatigue K. Chronic fatigue syndrome (CFS) or ME L. Dementia Other M. Digestive problems e.g. irritable bowel syndrome, Crohn’s disease N. Preventative i.e. to stay well, prevent ill-health, boost immune system O. Women’s health e.g. period problems, pregnancy, labour, fertility, symptoms of the menopause P. Cancer Q. Allergies e.g. hayfever, allergy to dust or animals R. Skin problems or infections e.g. eczema, psoriasis S. Cardiovascular problems e.g. heart disease T. Respiratory problems e.g. cough, colds or flu, asthma, bronchitis, emphysema U. Infections V. Diabetes W. Urinary tract disorders e.g. cystitis, kidney stones, incontinence X. Eye problems Y. Ear problems Z. Nervous system problems e.g. stroke, epilepsy, multiple sclerosis AA. Immune system problems e.g. HIV, autoimmune disease BB. Other (Please specify) CC. Don’t know
ASK ONLY THOSE WHO CODE A AT Q1 Q5 **Which, if any of these, describes how you came to use this complementary treatment?** A. My GP referred or recommended me B. Another NHS health professional (e.g. consultant) referred me C. My GP or another NHS health professional I was seeing provided the complementary treatment themselves D. Another complementary practitioner referred or recommended me E. I found them myself/ they were recommended by friend/family F. Other (please specify) G. Don’t know/can’t remember
ASK ONLY THOSE WHO CODE A AT Q1 Q6 **How was the treatment from this complementary practitioner paid for? Please select all that apply.** A. The NHS paid for all or part of it B. I or my family/friend paid for all or part of it C. Another organisation such as a charity paid for all or part of it D. My health insurance paid for all or part of it E. I don’t know/ don’t remember F. Other (please specify)
ASK ONLY THOSE WHO CODE B AT Q6 Q7 **On average, how much did you pay for each visit to this complementary practitioner?** A. Less than £10 B. Between £11 and £20 C. Between £21 and £30 D. Between £31 and £40 E. Between £41 and £50 F. Between £51 and £60 G. Between £60 and £100 H. Over £100 I. I don’t know/don’t remember J. Prefer not to say
ASK ONLY THOSE WHO CODE B AT Q1 Q8 **Please can you tell me why not?** A. I wasn’t seeking help for any health problems/ I didn’t have any health problems B. It hasn’t occurred to me C. I don’t know enough about it D. I don’t have time E. I don’t think it would help me F. I don’t believe in it G. I’m afraid of the side effects/ how it interacts with other medicines H. I am concerned about the practitioner not being qualified or regulated I. My GP/other NHS health professional has advised against it J. It is not available on the NHS K. It is not available locally L. I can’t afford to pay for it M. Other, (please specify) N. Prefer not to say O. Don’t know
ASK ALL Q9 **If you had a health problem you thought complementary or alternative medicine could help with, which, if any, of the following statements would encourage you to use it?** A. If it was free through the NHS or another organisation/ charity B. If it was partly paid for by the NHS or another organisation/ charity (I only had to pay a small amount) C. If the complementary practitioner was also an NHS health professional D. If the complementary practitioner was based in an NHS setting E. If the NHS regulated the complementary practitioner F. If my GP mentioned it might help G. If my GP referred me to the complementary practitioner H. If I thought it would enhance the care I was already receiving I. If I had to wait a long time for NHS care J. If my NHS treatment was not helping me K. None of the above L. Other (please specify) M. I would never use it N. Don’t know
ASK THOSE WHO CODE B AT Q1. DO NOT INCLUDE THOSE WHO CODE M AT Q9 Q10 **If you had a health problem you thought would improve from seeing a complementary or alternative practitioner, what is the most you would be willing to pay for each visit?** · £0 · Less than £10 · Between £11 and £20 · Between £21 and £30 · Between £31 and £40 · Between £41 and £50 · Between £51 and £60 · Between £60 and £100 · Over £100 · I don’t know · Prefer not to say
